# Inhibition of Lysine 63 Ubiquitination Prevents the Progression of Renal Fibrosis in Diabetic DBA/2J Mice

**DOI:** 10.3390/ijms22105194

**Published:** 2021-05-14

**Authors:** Paola Pontrelli, Francesca Conserva, Rossella Menghini, Michele Rossini, Alessandra Stasi, Chiara Divella, Viviana Casagrande, Claudia Cinefra, Mariagrazia Barozzino, Simona Simone, Francesco Pesce, Giuseppe Castellano, Giovanni Stallone, Anna Gallone, Francesco Giorgino, Massimo Federici, Loreto Gesualdo

**Affiliations:** 1 Department of Emergency and Organ Transplantation, University of Bari, 70124 Bari, Italy; francfbi@hotmail.com (F.C.); michelerossini@libero.it (M.R.); alessandra.stasi@uniba.it (A.S.); c.divella80@gmail.com (C.D.); cld.cinefra@gmail.com (C.C.); barozzino.mariagrazia@gmail.com (M.B.); simonasimone1976@gmail.com (S.S.); f.pesce81@gmail.com (F.P.); francesco.giorgino@uniba.it (F.G.); loreto.gesualdo@uniba.it (L.G.); 2 Department of Systems Medicine, University of Rome Tor Vergata, 00133 Rome, Italy; menghini@med.uniroma2.it (R.M.); viviana.casagrande@uniroma2.it (V.C.); federicm@uniroma2.it (M.F.); 3 Department of Medical and Surgical Sciences, University of Foggia, 71122 Foggia, Italy; giuseppe.castellano@unifg.it (G.C.); giovanni.stallone@unifg.it (G.S.); 4 Department of Basic Medical Sciences, Neurosciences and Sense Organs, University of Bari, 70124 Bari, Italy; anna.gallone@uniba.it

**Keywords:** DBA2/J mice, lysine 63 ubiquitination, microRNA, renal fibrosis, senescence, diabetes

## Abstract

Diabetic nephropathy (DN) is the most frequent cause of end-stage renal disease. Tubulointerstitial accumulation of lysine 63 (K63)-ubiquitinated (Ub) proteins is involved in the progression of DN fibrosis and correlates with urinary miR-27b-3p downregulation. We explored the renoprotective effect of an inhibitor of K63-Ub (NSC697923), alone or in combination with the ACE-inhibitor ramipril, in vitro and in vivo. Proximal tubular epithelial cells and diabetic DBA/2J mice were treated with NSC697923 and/or ramipril. K63-Ub protein accumulation along with α-SMA, collagen I and III, FSP-1, vimentin, p16^INK4A^ expression, SA-α Gal staining, Sirius Red, and PAS staining were measured. Finally, we measured the urinary albumin to creatinine ratio (uACR), and urinary miR-27b-3p expression in mice. NSC697923, both alone and in association with ramipril, in vitro and in vivo inhibited hyperglycemia-induced epithelial to mesenchymal transition by significantly reducing K63-Ub proteins, α-SMA, collagen I, vimentin, FSP-1 expression, and collagen III along with tubulointerstitial and glomerular fibrosis. Treated mice also showed recovery of urinary miR-27b-3p and restored expression of p16^INK4A^. Moreover, NSC697923 in combination with ramipril demonstrated a trend in the reduction of uACR. In conclusion, we suggest that selective inhibition of K63-Ub, when combined with the conventional treatment with ACE inhibitors, might represent a novel treatment strategy to prevent the progression of fibrosis and proteinuria in diabetic nephropathy and we propose miR-27b-3p as a biomarker of treatment efficacy.

## 1. Introduction

Diabetic nephropathy (DN) is a renal complication of diabetes that can lead to renal failure when untreated [1]. Several therapeutic options are currently available for the management of high blood pressure and hyperglycemia such as inhibitors of the renin angiotensin aldosterone system (RAAS), hypoglycemic drugs such as metformin, sodium glucose transporter 2 (SGLT-2) inhibitors, and glucagon-like peptide 1 (GLP-1) inhibitors. These molecules have been shown to exert beneficial effects in the context of renal damage and have also been shown to preserve renal function in animal models through their ability to inhibit pathways leading to renal fibrosis [2,3,4,5]. Moreover, results from recently published clinical trials have unveiled the effects of SGLT-2 inhibitors in reducing the risk of kidney failure as well as of death from cardiovascular causes or heart failure hospitalization in chronic kidney disease patients with or without type 2 diabetes [6]. However, no clinical data exploring renal fibrosis among other outcomes are currently available, and no targeted therapies have been developed to prevent or reduce the molecular process leading to renal fibrosis. 

Several molecular pathways are dysregulated as a consequence of chronic kidney exposure to hyperglycemia, thus representing pathogenic mechanisms in DN [7]. Over the last few years, our group has described the role of lysine 63 (K63) ubiquitylation in DN-related fibrosis [8]. The relevance of dysregulated protein ubiquitylation in DN is emerging. An increased expression of the E2 enzyme UBE2v1 (responsible for K63-linked polyubiquitin chains formation) is associated with epithelial to mesenchymal transition (EMT) of tubular cells, increased production of extracellular matrix, and progression of kidney fibrosis in diabetic patients [8,9]. This process is involved in autophagy-induced cell death [10] and can be monitored by dosing specific urinary miRNAs, particularly miR-27b-3p and miR-1228-3p, whose expression levels inversely correlate with renal fibrosis [11]. However, the beneficial effects of inhibiting this pathway in vivo remain to be explored.

Another mechanism responsible for kidney fibrosis in DN involves the accelerated cellular senescence of tubular cells [12,13]. DcR2, a transmembrane receptor of tumor necrosis factor–related apoptosis-inducing ligand (TRAIL) belonging to the tumor necrosis factor receptor superfamily [14], represents a potential target to modulate the induction of cellular senescence in tubular cells. It has also been demonstrated that hyperglycemia dramatically increases the renal expression of the cyclin-dependent kinase (CDK) inhibitor p21, thus inducing an increase in the senescence of tubular epithelial cells [15]. However, the role of the different mechanisms mediating kidney aging in diabetes remains to be fully clarified. 

Among the different models to study diabetes and diabetic complications, streptozotocin (STZ)-treated DBA/2J mice interestingly share common transcriptional networks with established importance in human DN [16,17]. Specifically, when treated with STZ, a compound with toxic effects on the insulin-producing pancreatic beta-cells resulting in hyperglycemia, DBA/2J mice typically develop albuminuria, mesangial expansion, and GFR decline [16]. 

In this study, we aimed to investigate whether the in vitro and in vivo inhibition of K63-linked polyubiquitin chains accumulation, using the specific E2 complex inhibitor compound NSC697923, alone and in combination with the RAAS inhibitor ramipril, has beneficial effects in terms of preventing kidney fibrosis and reducing albuminuria. We also aimed to evaluate if circulating miRNAs can be used as specific biomarkers to monitor the efficacy of the different treatments.

## 2. Results

### 2.1. Hyperglycemia-Induced K63 Protein Ubiquitination and Epithelial to Mesenchymal Transition in HK2 Cells Are Not Reduced by Ramipril Alone but Only by the Association with NSC697923

Cell viability on tubular cells revealed the absence of cytotoxic effects by the different treatments (Supplementary Figure S1). Since ACE-inhibitors including ramipril may provide renoprotection despite their inability to completely prevent renal fibrosis, we first investigated whether ramipril was able to modulate hyperglycemia-induced K63 ubiquitination. 

**Figure 1:**
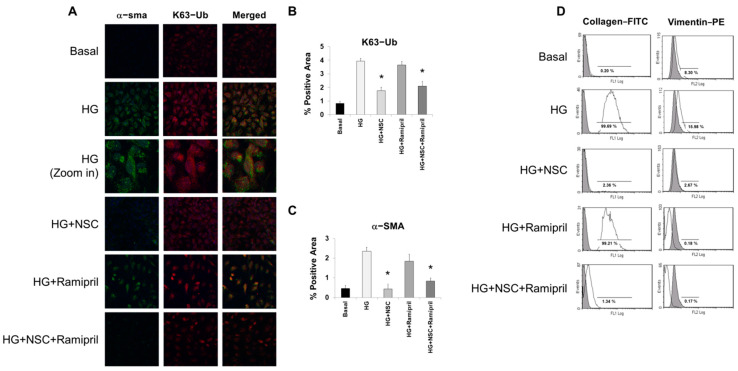
NSC697923, alone and in combination with ramipril, but not ramipril alone, reduced EMT in tubular cells. (**A**) Immunofluorescence (IF) depicting α-sma expression (green channel), K63 ubiquitinated proteins distributions (red channel), and the merged signal in HK2 cells under the indicated treatments. Figure magnification 63X. (**B**,**C**) IF quantification for K63-Ub (**B**) and α-sma (**C**). Expression was quantified as the percentage of positive area. * *p* < 0.0001 vs. HG. (**D**) Flow cytometry analysis on collagen I and vimentin. % of positive cells in the different experimental conditions is indicated in the figure panels. Results are representative of three independent experiments.

In HK2 cells, ramipril treatment alone did not affect hyperglycemia-induced K63 ubiquitination, while this process was significantly reduced by pre-incubation with NSC697923, both alone and in association with ramipril (Figure 1A,B). In addition, ramipril alone did not modify the expression of α-SMA in HK2 cells grown in high glucose, suggesting that ACE inhibition alone is not sufficient to block hyperglycemia-induced epithelial to mesenchymal transition of tubular cells. Interestingly, when HK2 cells were grown in high glucose with the addition of NSC697923, either alone or in association with ramipril, α-SMA expression was significantly reduced and comparable to the control (Figure 1A–C). Flow cytometry analysis showed a marked increase of collagen I following 48 h of stimulation with high glucose when compared to the control, indicating that EMT actively contributes to the synthesis of extracellular matrix components (Figure 1D). In addition, the mesenchymal marker vimentin was also increased following high glucose stimulation. Ramipril alone was able to reduce vimentin expression while it had no effect in the suppression of collagen I synthesis. Remarkably, NSC697923 alone or in combination with ramipril completely restored the epithelial phenotype (Figure 1D).

### 2.2. NSC697923, Alone and in Combination with Ramipril, but Not Ramipril Alone, Reduced K63-Ub Proteins Accumulation, and Renal Fibrosis in Diabetic Mice

DBA/2J mice were used as our in vivo model of DN. Animals were initially injected with either STZ or PBS, and then treated with ramipril and NSC697923, alone and in combination. After sacrifice, kidneys were processed to investigate the in vivo pattern of K63 ubiquitination and to characterize renal fibrosis through Sirius Red and PAS staining as well as through the expression of collagen III and FSP-I. 

**Figure 2:**
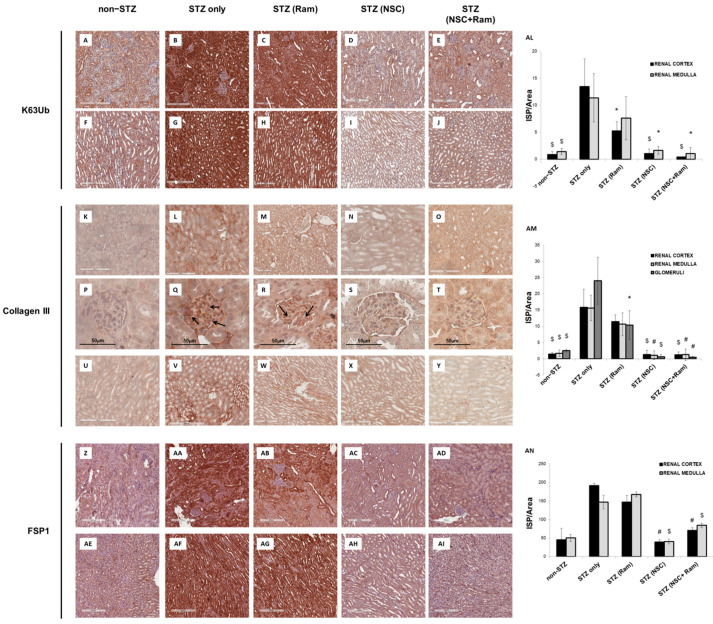
NSC697923, alone and in combination with ramipril, reduced K63-ubiquitination, collagen III expression. and FSP-1 expression in the renal cortex and medulla of STZ-treated DBA/2J mice. (**A**–**J**,**AL**). Immunoperoxidase staining against K63-ubiquitinated proteins on renal formalin-fixed paraffin-embedded section of DBA/2J mice. (**K**–**Y**,**AM**) Collagen expression evaluated by immunoperoxidase staining against collagen III. Black arrows indicate Collagen III deposits within glomerular capillaries (**Z**–**AI**,**AN**). Staining of fibroblasts derived from EMT evaluated by immunoperoxidase staining against FSP-1. All experiments were performed on five different groups of animals, each group including at least four different animals. Non-diabetic animals are indicated as non-STZ; diabetic animals that did not receive any other compound are indicated as STZ-only; diabetic animals treated with either ramipril, NSC697923, or the combination, are indicated respectively as STZ (Ram), STZ (NSC), and STZ (NSC + Ram). Mean intensity of strong positive related to the area analyzed was measured for K63-ubiquitination, collagen III, and FSP-1 immunohistochemistry. Error bars indicate standard deviation. * *p* < 0.05 vs. STZ only; ^$^ *p* < 0.005 vs. STZ only; ^#^ *p* < 0.0005 vs. STZ only.

The analysis of K63-ubiquitinated proteins in STZ-induced diabetic DBA/2J mice demonstrated a significant accumulation of these proteins in both the renal cortex (Figure 2B) and medulla (Figure 2G) of diabetic mice compared to the control animals (Figure 2A,F). Notably, this increase was mostly confined to the tubular compartment and less evident in glomeruli, suggesting an increased uptake of K63-ubiquitinated proteins by proximal tubular cells, as previously described in human DN [8]. While treatment with ramipril resulted in a non-significant reduction of K63-ubiquitinated proteins neither in the renal cortex (Figure 2C) nor in the medulla (Figure 2H), the selective K63 ubiquitination inhibitor NSC697923 significantly diminished K63 ubiquitination when used both alone (Figure 2D,I) and in combination with ramipril (Figure 2E,J). Nondiabetic mice treated with ramipril or NSC697923, alone and in combination, did not show any significant difference in the level of K63-ubiquitinated proteins (Supplementary Figure S2). 

The effects of both NSC697923 and ramipril on the development of tubule-interstitial fibrosis within the renal cortex, renal medulla, and glomeruli was investigated by collagen III and FSP-I immunohistochemistry as well as Sirius Red and Fast Green staining, as previously described [18], and PAS staining. For the quantification of each staining associated with computerized image analysis, the perivascular regions as well as the limits of each biopsy were excluded from the analysis of the cortical and medullary compartments, while the Bowman’s capsule was excluded when analyzing the glomerular area. Diabetic mice showed an increased amount of collagen III deposition, both in the renal cortex, at the tubulo-interstitial (Figure 2L) and glomerular (Figure 2Q) levels, and in the renal medulla (Figure 2V), when compared with non-diabetic mice (Figure 2K,P,U, respectively). Interestingly, packed collagen III fibers were accumulated in the glomerular mesangium (Figure 2Q), suggesting the development of renal damage comparable to human DN.

In diabetic mice, treatment with ramipril resulted in a slight reduction in collagen III expression at the tubulo-interstitial level and medulla (Figure 2M,W, respectively), which was significant only at the glomerular level (Figure 2R) when compared to untreated diabetic mice (Figure 2P). Interestingly, NSC697923 alone (Figure 2N,S,X) or in combination with ramipril (Figure 2O,T,Y) preserved the integrity of the renal parenchyma by significantly reducing collagen III deposits at both the tubulo-interstitial and glomerular levels and in the medullary region, so that levels were comparable to those in non-diabetic mice. Control mice, both untreated (Figure 2K,P,U) and treated (Supplementary Figure S3), showed similar amounts of collagen III fibers in all compartments of renal parenchyma.

Additionally, FSP-1 expression, another myofibroblast marker, almost absent in non-diabetic mice both in the cortex (Figure 2Z) and in the medulla (Figure 2AE), was significantly increased in diabetic mice (Figure 2AA,AF). Ramipril treatment did not significantly reduce FSP-1 expression in both the cortex (Figure 2AB) and in the medulla (Figure 2 AG). NSC697923 both alone (Figure 2AC,AH) or in combination with ramipril (Figure 2AD,AI) significantly reduced FSP-1 expression in the tubular compartment in the cortex (Figure 2AC,AD, respectively) and in the medulla (Figure 2AH,AI, respectively) once again to levels comparable to those in non-diabetic mice.

**Figure 3:**
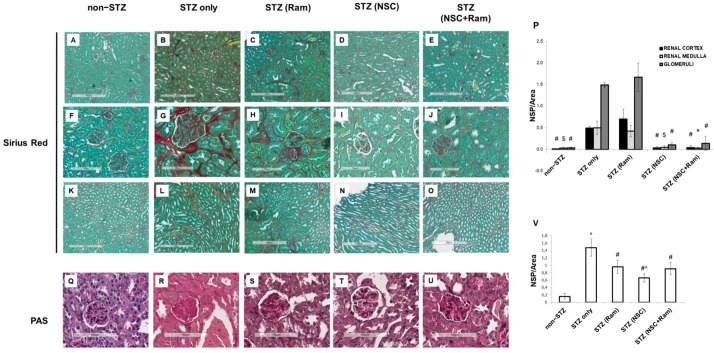
NSC697923 effect, alone and in combination with ramipril, on Sirius Red staining and PAS staining in the renal cortex and medulla of STZ-treated DBA/2J mice. (**A**–**O**,**P**). Collagen expression evaluated by Sirius Red staining on five different groups of animals, each group including at least four different animals. Sirius Red staining was quantified by analyzing the mean number of strong positives on the area analyzed. Error bars indicate standard deviation. (**Q**–**U**,**V**). Representative PAS staining of glomeruli from five different groups of animals, each group including three different animals. PAS staining was quantified analyzing the mean number of strong positive on the area analyzed. Error bars indicate standard deviation. Non-diabetic animals are indicated as non-STZ; diabetic animals that did not receive any other compound are indicated as STZ-only; diabetic animals treated with either ramipril, NSC697923, or the combination are indicated respectively as STZ (Ram), STZ (NSC), and STZ (NSC + Ram). * *p* < 0.05 vs. STZ only; ^$^ *p* < 0.005 vs. STZ only; ^#^ *p* < 0.0005 vs. STZ only; ° *p* < 0.0001 vs. non-STZ; ^ *p* < 0.005 vs. STZ (Ram) and STZ (NSC+Ram).

Sirius Red/Fast Green staining was used to confirm the sclerosing condition of diabetic kidney damage. As expected, in diabetic animals, large amounts of tightly packed collagen fibers were found in the renal cortex, at both the tubule-interstitial (Figure 3B) and glomerular (Figure 3G) levels and in the renal medulla (Figure 3L). No significant difference in collagen fiber content was observed following treatment with ramipril compared to the control in all renal compartments (Figure 3C,H,M,P). Remarkably, the use of NSC697923 significantly reduced the accumulation of collagen fibers in all renal compartments (Figure 3D,I,N), and this decrease was also achieved when NSC697923 was used in combination with ramipril (Figure 3E,J,O,P). Healthy animals, both untreated (Figure 3A,F,K) and treated (Supplementary Figure S4) displayed comparable amounts of collagen fibers and red-stained fibrillary elements that were constitutively found on larger arterial walls and along the Bowman’s capsule.

We also performed PAS staining in order to evaluate mesangial matrix accumulation. Diabetic mice demonstrated an increased mesangial matrix deposition (Figure 3R,V) when compared to control non diabetic mice (Figure 3Q). Ramipril treatment (Figure 3S) as well as NSC697923 treatment both alone (Figure 3T) and in association with ramipril (Figure 3U) determined a significant reduction in mesangial matrix deposition, however, NSC697923 treatment alone determined a higher degree of reduction when compared to both ramipril and the combination. 

Collectively, these data indicate that NSC697923 alone or in association with ramipril can counteract the mechanisms that promote glomerulosclerosis and tubular-interstitial fibrosis in experimental diabetic nephropathy.

### 2.3. Effect of NSC697923, Alone or in Combination with Ramipril, on Albuminuria and on the Concentration of Urinary miR-27b-3p in Diabetic Mice

**Figure 4:**
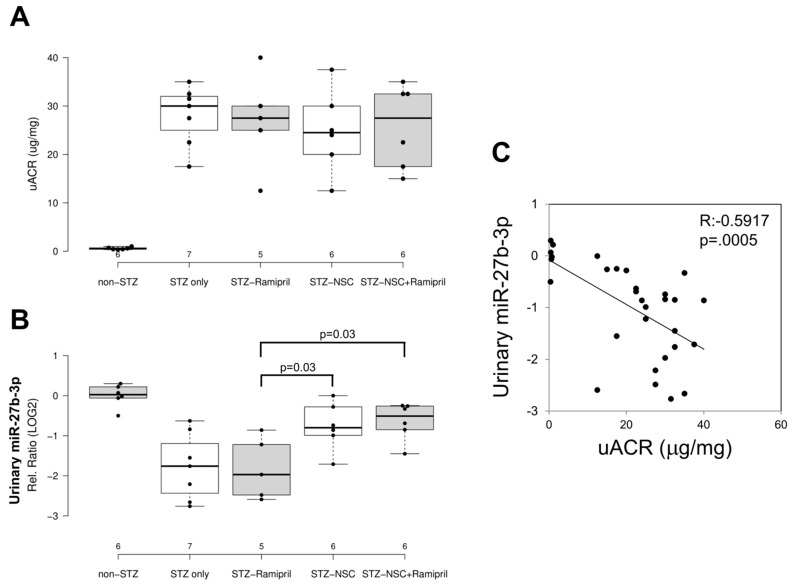
Effects of NSC697923, alone and in association with ramipril, on uACR (**A**) and urinary miR-27b-3p levels (**B**) and correlation among both (**C**). (**A**) Boxplot depicting urinary albumin to creatinine ratio excretion in non-diabetic animals (non-STZ), untreated diabetic animals (STZ-only), diabetic animals treated with ramipril (STZ-Ramipril), diabetic animals treated with NSC697923 (STZ-NSC), or diabetic animals treated with both ramipril and NSC697923 (STZ-NSC + Ramipril). Number of mice analyzed is reported in the graph. Error bars indicate standard deviation. (**B**) miR-27b-3p expression in DBA/2J urinary samples. RNA samples were isolated from 24 h urines. miR-27b-3p was measured using real-time PCR as described in the Methods; results are depicted as LOG2 in the boxplot and were normalized to the relative levels of the exogenous Cel-39 spike-in. Number of mice analyzed is reported in the graph. (**C**) Correlation between uACR and urinary miR-27b-p. R value and *p* value are reported in the image.

In order to evaluate the effects of ramipril and NSC697923 on STZ-induced albuminuria in diabetic mice, we performed quantification of urinary albumin and normalization with urinary creatinine (Figure 4A). As expected, the mean urinary albumin to creatinine ratio (uACR) was higher in the diabetic compared to control mice. Moreover, NSC697923, better than ramipril alone and the combination of both, was able to mitigate STZ-induced albuminuria, however no statistical significance was observed, probably due to the reduced number of animals available for this analysis. 

To evaluate the effect of NSC697923, alone or in combination with ramipril, on the expression of candidate biomarkers of diabetes-induced renal injury in vivo, we assessed the urinary levels of miR-27b-3p in our animal model. We focused on the analysis of miR-27b-3p, since this miRNA, unlike miR-1228-3p, is evolutionarily conserved in mice. Interestingly, miR-27b-3p was significantly downregulated in diabetic mice (STZ-only) compared to the control group (non-STZ) (Figure 4B) as observed in human samples [11]. Treatment with ramipril did not affect miR-27b-3p levels in urine of DBA/2J diabetic mice, however, the treatment with NSC697923, alone and in combination with ramipril, was associated with the restoration of miR-27b-3p levels.

Interestingly, we observed a moderate but very significant inverse correlation between uACR and urinary miR-27b-3p (Figure 4C), thus further supporting the role of miR-27b-3p as a biomarker of renal function decline in diabetes.

### 2.4. NSC697923, Alone and in Combination with Ramipril, Modulates Cellular Senescence, Both In Vitro and In Vivo

Premature senescence is a key cellular event in renal fibrosis and is associated with the progression of diabetic nephropathy [12,14,19]. 

**Figure 5:**
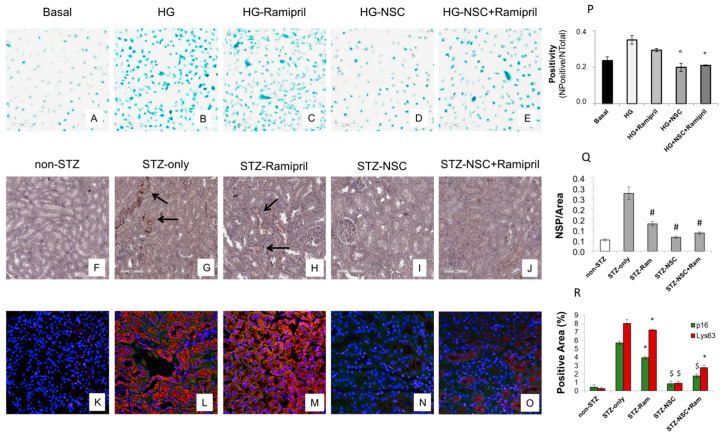
Treatment with NSC697923 modulated cellular senescence and renal expression of p16^INK4A^ in vitro in RPTEC cells and in vivo in DBA/2J mice. (**A**–**E**,**P**) SA-β Gal staining was obtained as described in the Methods in RTEC under basal conditions, exposed to high glucose (HG), and treated with ramipril, NSC697923 (NSC), or both. The ratio of cells positive for SA-β-gal activity was calculated by examining five not overlapping fields per condition. The results are presented as the mean ± SD of three independent experiments. (**F**–**O**,**Q**,**R**) P16^INK4A^ was measured using both immunoperoxidase staining (**A**–**E**,**K**) and immunofluorescence (**F**–**J**,**L**) in renal formalin-fixed paraffin-embedded section of non-diabetic animals (non-STZ), untreated diabetic animals (STZ-only), diabetic animals treated with ramipril (STZ-Ramipril), diabetic animals treated with NSC697923 (STZ-NSC), and diabetic animals treated with both ramipril and NSC697923 (STZ-NSC + Ramipril). At least four mice per group were used. The lower panel shows immunofluorescence of p16^INK4A^ in the green channel and K63-ubiquitinated proteins in the red channel. Figure magnification 63X. The immunoperoxidase staining was quantified as the number of strong positives (NPS) on the area analyzed; P16^INK4A^ and K63-ubiquitinated protein expression in immunofluorescence was quantified as a percentage of the positive area on the area analyzed. Histograms are indicative of mean values while error bars are indicative of the standard deviation. ° *p* < 0.05 vs. HG; * *p* < 0.05 vs. STZ only; ^$^ *p* < 0.005 vs. STZ only; ^#^ *p* < 0.0005 vs. STZ only.

Therefore, we evaluated whether NSC697923 might affect hyperglycemia-induced tubular cell senescence in vitro. To this purpose, we stimulated RPTEC with high glucose for 48 h both in the absence (Figure 5B) and in the presence of ramipril (Figure 5C), or NSC697923 (Figure 5D), or the combination (Figure 5E). Interestingly, high glucose stimulated-RPTEC acquired a senescent phenotype as determined by significant increase in SA-β-GAL positivity compared to the basal conditions (Figure 5A,B,P). Interestingly under hyperglycemic conditions in the presence of ramipril, RPTEC cells did not show a significant reduction in hyperglycemia-induced SA-βGAL positivity (Figure 5C,P), while NSC697923 both alone (Figure 5D,P) and in association (Figure 5E,P) significantly reduced cellular senescence and SA-βGAL positivity (Figure 5P).

Moreover, we investigated the expression of the senescent marker p16^INK4A^ and its potential association with the accumulation of K63-ubiquitinated proteins in vivo.

Non-diabetic mice displayed few p16^INK4A^ positive tubular cells in renal parenchyma (Figure 5F). In contrast, p16^INK4A^ staining was greatly increased in diabetic mice (Figure 5G). NSC697923, alone (Figure 5I) and in combination with ramipril (Figure 5J), significantly reduced the expression of the senescence-related p16^INK4A^ marker (Figure 5P). Ramipril treatment induced a significant reduction of p16^INK4A^ expression when compared to untreated diabetic mice, but this effect was less evident compared to NSC697923 (Figure 5H,Q).

To further evaluate the role of K63-ubiquitinated protein accumulation in tubular senescence, we performed a double immunofluorescence staining for p16^INK4A^ and K63-ubiquitinated proteins in all animal groups. The expression of K63-ubiquitinated proteins colocalized with p16^INK4A^-positive tubules in the diabetic mice (Figure 5L), and no significant differences in the co-staining were observed following treatment with ramipril (Figure 5M,R). Remarkably, the use of NSC697923 alone (Figure 5N) or in combination with ramipril (Figure 5O) significantly reduced K63-ubiquitinated proteins/p16^INK4A^ double positive cells, indicating diminished renal senescence (Figure 5R). Collectively, these data indicate that NSC697923 alone or in association with ramipril could interfere with diabetes-induced senescence and prevent kidney fibrosis and progression of kidney damage.

## 3. Discussion

In the present paper, we demonstrated that (i) tubular epithelial cells grown in high glucose were characterized by increased α-SMA, collagen I, vimentin, and K63 ubiquitination and that treatment with NSC697923 (alone and in association with ramipril), but not with ramipril alone, was effective at blocking K63-induced epithelial to mesenchymal transition in vitro; (ii) diabetic DBA/2J mice showed accumulation of K63-ubiquitinated proteins in the renal tubular-interstitial compartment and were also prone to developing renal damage due to increased fibrosis; (iii) diabetic DBA/2J mice treated with ramipril showed a mild reduction of K63-ubiquitinated proteins and subsequent fibrosis, suggesting that this compound cannot completely block these molecular changes; (iv) the selective inhibitor of K63 ubiquitination, NSC697923, both alone and in association with ramipril, but not ramipril alone, not only suppressed K63 ubiquitination, but also collagen deposition and glomerulosclerosis in vivo; (v) NSC697923 treatment seemed to also be effective in the amelioration of the albumin to creatinine ratio in diabetic DBA/2J mice; (vi) the levels of urinary miR-27b-3p in diabetic DBA/2J mice, which were reduced in diabetic mice compared to non-diabetic controls, could be restored by NSC697923, alone and in combination with ramipril and significantly correlated with uACR; and (vii) NSC697923, alone and in combination with ramipril, was able to block the high-glucose induced cellular senescence and p16^INK4A^ upregulation.

DN is a serious complication of diabetes that can lead to end-stage renal disease [1]. New therapies to slow down the progression of diabetic kidney disease are thus eagerly needed. The standard treatment regimen for patients with DN includes glucose-lowering agents along with compounds that modulate the renin-angiotensin (RAS) system such as ramipril [20]. Despite optimal treatment and the proven efficacy demonstrated by a novel class of antidiabetic drugs such as SGLT-2 inhibitors in preventing the decline of renal function [6], the preservation of renal integrity in diabetic patients remains challenging, and the disease can ultimately lead to kidney fibrosis. ACE inhibition has potential anti-fibrotic effects in murine models of unilateral ureteral obstruction and progressive renal fibrosis [21] as well as in diabetic mice by inhibiting hyperglycemia-induced growth factor production and subsequent fibroblast activation [22]; however, other mechanisms can contribute to the development of renal fibrosis in diabetic patients, and it is unknown whether ACE inhibitors or other novel drugs such as SGLT-2 inhibitors may modulate these processes. Among the several mechanisms involved in kidney fibrosis, K63 ubiquitination promotes epithelial to mesenchymal transition and progression of tubular fibrosis in DN patients [8]. Inhibition of K63 ubiquitination in vitro can block both epithelial to mesenchymal transition and apoptosis of tubular cells induced by autophagy [10], however, no evidence has been described regarding the effect of this inhibition in vivo.

NSC697923 is a small molecule that inhibits the activity of the ubiquitin-conjugating (E2) enzyme Ubc13-Uev1A, involved in the synthesis of K63-linked polyubiquitin chains. NSC697923 was first described in 2012 by Pulvino et al., who proposed it as a possible therapeutic agent to counteract diffuse large B-cell lymphoma [23]. Its role in the treatment of neuroblastoma [24], colorectal cancer [25], and melanoma [26] has been also explored; however, to our knowledge, this is the first study where this compound was used for the treatment of DN in vivo.

Interestingly, we observed that the reno-protective effects of NSC697923 were not only limited to the prevention of tubular-interstitial fibrosis via reduction of lysine 63 ubiquitinated protein accumulation and FSP-1 expression, but also to the decreased collagen III deposition in the mesangium with the reduction in the PAS positive mesangial matrix. Hyperglycemia can induce α-sma and collagen IV production in mesangial cells and ubiquitination seems involved in this process. In particular, ubiquitination and degradation of lysine-specific demethylase 3A and the downstream modulation of the TGF-β1/Smad2/Smad3 signaling induced by the plant extract magnoflorine attenuates DN and suppresses inflammatory responses and fibrosis in mesangial cells both in vitro and in vivo [27]. Mesangial collagen synthesis in DN is also modulated by the E3 ubiquitin ligase, TRIM13, by promoting ubiquitination and degradation of C/EBP homologous protein [28]. No data are available regarding the specific role of lysine 63 ubiquitination in mesangial cells under hyperglycemic conditions and further investigation is needed. K63-linked chains can modulate non-proteolytic functions in at least four pathways: DNA damage repair, intracellular signaling, intercellular trafficking, and ribosomal biogenesis [29], all potentially involved in α-sma, FSP-1, and collagen deposition in these cells.

We observed that NSC697923 treatment alone also showed a reduction in uACR in diabetic mice. Although this reduction did not reach a statistical significance, perhaps due to the reduced number of animals included in the study and the great variability observed among them, however, this trend is in line with the reduction in glomerular damage assessed by the reduction of both collagen III deposition and PAS staining.

Increased fibrosis due to K63 ubiquitination is linked to the reduced expression of miR-1228-3p and miR-27b-3p in the human urine samples [11]. In the present study, we confirmed the involvement of miR-27b-3p, the only one conserved among humans and mice, as a biomarker correlated with the kidney fibrosis associated with diabetes-induced K63 ubiquitination. Treatment with NSC697923, both alone and in association with ramipril, resulted in restored miRNA-27b-3p levels, which returned similar to those detected in non-diabetic mice, while ramipril alone did not modify miRNA levels in diabetic mice. The role of this miRNA in the pathogenesis of DN has been suggested by Wang et al., who demonstrated its downregulation in diabetic mice and its link to increased epithelial to mesenchymal transition [30]. Thus, in the present manuscript, miR-27b-3p has emerged as a biomarker that can also be used to monitor therapy efficacy in terms of fibrosis reduction. Moreover, we also demonstrated a significant correlation between miR-27b-3p urinary levels and uACR, thus supporting the role of this particular miRNA as a biomarker for the progression of renal damage in diabetes.

It is well known that aging is strongly associated with kidney disease. End stage renal disease (ESRD) is common in the elderly [1] and diabetes is a strong risk factor for ESRD, since chronic glucose exposure promotes oxidative stress and cell senescence in the kidney [31]. Moreover, it has been described that increased p16^INK4A^ expression correlates with tubular atrophy and interstitial fibrosis [32], and thus p16^INK4A^ expression might be relevant to monitor renal senescence. We found that NSC697923, both alone and in association with ramipril, could significantly block cellular senescence by reducing p16^INK4A^ expression in vivo. Moreover, in vitro treatment with NSC697923 both alone and in association with ramipril, reduced SA-β-Gal positivity, thus indicating a specific effect on cellular senescence in tubular cells. Thus, NSC697923 might also be considered as a “senolytic drug”. Senolytic compounds are a class of molecules with growing importance as they selectively clear senescent cells and can alleviate multiple chronic diseases in experimental animals [33]. Further experiments will be needed to clarify the molecular pathways matching this cellular senescence with K63 protein ubiquitination.

## 4. Materials and Methods

### 4.1. Cell Cultures

HK2, an immortalized proximal tubular epithelial cell line from normal adult human kidney, was purchased from American Type Culture Collection (Rockville, MD, USA, RRID: CVCL_0302). Cells were cultured in DMEM with 1000 mg glucose/L supplemented with 10% fetal bovine serum, 100 U/mL penicillin, 100 mg/mL streptomycin, and 2 mM l-glutamine (Sigma-Aldrich, St. Louis, MO, USA). This condition was regarded as basal (low glucose: 5.5 mM). For passage, confluent cells were washed twice with PBS, detached from the culture flask with 0.5% trypsin/0.02% EDTA in PBS, and plated in complete medium with or without 24.5 mM D-glucose (high glucose). Cells were then incubated at 37 °C with 5% CO_2_. 

Human RPTECs were purchased from Lonza (Lonza, Basel, Switzerland). RPTECs were maintained in renal epithelial cell growth basal medium REBM supplemented with rhEGF (recombinant human EGF), transferrin, insulin, hydrocortisone, epinephrine, triidothyronine, and fetal bovine serum (FBS). 

### 4.2. Cells and Tissue Immunofluorescence Staining and Confocal Microscopy Analysis 

K63 ubiquitination and α-SMA expression were measured in HK2 cells through indirect immunofluorescence and confocal microscopy analysis. Cells were plated on a coverslip at the density of 5 × 10^4^ and incubated with a final concentration of 30 mM D-glucose for 48 h. NSC697923 inhibitor [1 mM] and ramipril [10 μM] were preincubated alone and in combination 1 h before adding D-glucose. HK2 cells were then fixed with 4% paraformaldehyde and treated with 0.2% Triton-X 100/PBS. p16 protein expression was evaluated on kidney mouse tissues. Cells and tissues were incubated in blocking buffer and then with the anti-ubiquitin, K63-specific antibody (Merck KgaA, Darmstadt, Germany) or anti-α-SMA antibody (Abcam, Cambridge, UK) or p16INK4a antibody (Abcam). Immune complexes were identified by incubating cells or tissue with the secondary antibody (Alexa Fluor 555 or Alexa Fluor 488; Thermo Fisher Scientific, Waltham, MA, USA). Samples were counterstained with TO-PRO-3 (Thermo Fisher Scientific), mounted in Fluoromount (Vector Laboratories, Burlingame, CA, USA), and sealed. The negative control was obtained omitting the primary antibodies. The specific fluorescence was analyzed by confocal laser scanning microscopy with the TCS SP2 or the Leica TCS SP8, (Leica, Wetzlar, Germany), equipped with argon-krypton (488 nm), green neon (543 nm), and helium neon (633 nm) lasers. Images were recorded with the Leica imaging software and quantified by the ImageJ software as described previously [10].

### 4.3. Flow Cytometry on HK2 Cells

HK2 cells were stained with the following monoclonal antibodies (mAbs) for flow cytometry analysis (FACS): PE-conjugated anti-vimentin (Miltenyi Biotec, Bergisch Gladbach, Germany), FITC-conjugated anti-collagen (Millipore, Millimarck, Germany) (or their corresponding isotype controls). Intracellular staining was performed by fixation and permeabilization with the IntraPrep Kit (Beckman Coulter, Milan, Italy). Then, cells were incubated for 20 min with the antibodies in the dark at room temperature, washed twice, and resuspended in FACS buffer. 

Stained cells were then acquired by a FC500 (Beckman Coulter) flow cytometer and analyzed with Kaluza software. Three independent experiments were performed. The area of positivity was determined by using an isotype-matched mAb, and in total, 10^4^ events for each sample were acquired. 

Cell viability of RPTEC cells in different conditions and under different treatments was measured using Annexin-V and 7AAD staining, according to the manufacturer’s protocol (Beckman Coulter).

### 4.4. SA-β-Gal Test 

Senescence-associated SA-β-gal staining on RPTEC was performed as described in the manufacturer’s protocol (Cell Signaling Technology, Danvers, MA, USA). Briefly, RPTEC cells were plated in 2-well Chamber slides at a cell density of 75.000 cell/well in a total volume of 1 mL DMEM medium containing 10% FBS, 1% L-glutamin, and 1% pen/strep and incubated for 48 h. Before staining, cells were washed. After washing with PBSin, then PBs, cells were fixed in 4% paraformaldehyde for 15 min and stained with freshly prepared SA-β-gal solution. Cells were then incubated at 37 °C overnight in a dry incubator. The following day, slides were mounted using glycergel mounting medium (Dako, Glostrup, Denmark) before assessing the development of a blue color. Specifically, slides were then scanned using the Aperio ScanScope (Leica Biosystems, Wetzlar, Germany) and the number of SA-β-gal-positive cells was determined by using the Aperio ImageScope software (Leica Biosystems) along with the positive pixel count algorithm v9. Results were reported as NP/Ntotal were NP represents the number of positive pixels and Ntotal represents the number of total pixels.

### 4.5. DBA/2J Animal Model

Animal studies were approved by the University of Tor Vergata Animal Care and Use Committee. The diabetic DBA/2J animal group was obtained with a total of five subsequent daily intraperitoneal streptozotocin (STZ) injections on 8-week old animals at the dose of 45 mg/kg in sodium citrate (pH 4.5). Control animals were injected with sodium citrate (pH 4.5) at the same age. DBA/2J mice were maintained in our animal facility (12 h light/dark cycle; 22 ± 1 °C, 50 ± 5% humidity) and fed ad libitum with standard laboratory chow (Mucedola s.r.l.). At the end of each experiment, mice were sacrificed by cervical dislocation, and tissues were collected for biochemical and morphological analyses. At the end of each experiment, individual mice were placed in metabolic cages for 24 h to obtain urine collections. The efficacy of STZ treatment was confirmed through the evaluation of blood glucose level changes before and after treatment to verify that all STZ-treated animals reported glucose levels above 250 mg/dL. Those STZ-treated animals where then divided in the different treatment groups and the number of control animals were defined consequently. Drugs were then tested, either alone or in combination, on both groups through six weekly injections starting five weeks following the first STZ or sodium citrate injection. The following drug treatments were administered over a total time of six weeks: three weekly intraperitoneal injections of phosphate buffered saline (PBS) on four STZ-treated animals and five sodium citrate-treated animals; three weekly intraperitoneal injections of 2% dimethyl sulfoxide (DMSO) on four STZ-treated animals and five sodium citrate-treated animals; three weekly intraperitoneal injections of the NSC697923 compound (3 mg/kg) diluted in PBS on seven STZ-treated animals; three weekly intraperitoneal injections of ramipril (2 mg/kg) in 2% DMSO on seven STZ-treated animals and five sodium citrate-treated animals; three weekly intraperitoneal injections of NSC697923 (3 mg/kg) diluted in PBS and ramipril (2 mg/kg) in 2% DMSO on six STZ-treated animals and five sodium citrate-treated animals.

All animals were sacrificed 11 weeks following the first STZ or PBS treatment and the following samples were harvested: 24 h urine, liver, heart, and kidney. Alongside, the following clinical data were recorded for each animal: total weight and blood glucose before STZ or PBS treatment and at sacrifice, urine volume and glucose concentration in 24 h collections before sacrifice.

### 4.6. Sirius Red/Fast Green Staining and PAS Staining on Formalin-Fixed Paraffin-Embedded DBA/2J Kidney Sections

Four-μm-thick sections of formalin-fixed paraffin-embedded DBA/2J kidney sections were deparaffinized and rehydrated with alcohol. Slides were then washed with distilled water and incubated in 0.04% Fast Green for 15 min at room temperature. Slides were then washed with distilled water and incubated in 0.1% Fast Green and 0.04% Sirius Red in saturated picric acid for 30 min. Then, they were dehydrated and mounted with DPX Mounting. Collagen fibers appeared red, while the non-collagen proteins were green. Images were acquired by the Aperio ScanScope CS2 device (Aperio Technologies, Vista, CA, USA), and the entire digital slides were analyzed with ImageScope V12.1.0.5029 (Aperio Technologies). Peri-vascular regions, the Bowman’s capsule, and the limits of each biopsy section were excluded from the analysis by drawing tools. Red collagen fibers were quantified by selecting the appropriate analysis algorithm that was generated by the renal pathologist (Image Analysis algorithm, Aperio). Results were expressed as the ratio of number of strong positive (NSP) over the total area analyzed (NSP/Area). Quantification of glomerular matrix deposition was performed on PAS stained slides. PAS positive matrix was quantified on each glomerulus (excluding the Bowman’s space and capsule) by selecting the appropriate analysis algorithm that was generated by the renal pathologist (Image Analysis algorithm, Aperio). Average values for each animal were then calculated. 

### 4.7. Immunohistochemistry

For the immunohistochemical evaluation of collagen III (Abcam), fibroblast specific protein-1 (FSP-1, Abcam), Lys63-ubiquitinated proteins (Merck KgaA), and p16INK4A (Abcam), 4 µm-thick paraffin-embedded renal tissue underwent deparaffination and heat-mediated antigen retrieval (citrate buffer, pH = 6.00). After epitope unmasking, the slides were incubated with H_2_O_2_ (3%) and then with Triton (0.25%), protein block solution (Dako), and the primary antibody. Primary antibodies were detected by the Peroxidase/DAB Dako Real EnVision Detection System, according to the manufacturer’s instructions (Dako). Renal sections were counterstained with Mayer hematoxylin and mounted with glycerol (Dako Cytomation, Glostrup, Denmark). Negative controls were prepared with isotype control Ab. Images were acquired by Aperio ScanScope CS2 device (Aperio Technologies) and signals analyzed with ImageScope V12.1.0.5029 (Aperio Technologies). Both collagen III and Lys63-ubiquitinated and p16INK4A protein staining was quantified using the cytoplasm algorithm that was generated by the pathologist (IHC Cytoplasm Image Analysis algorithm, Aperio). Peri-vascular regions, the Bowman’s capsule, and the limits of each biopsy section were excluded from the analysis by drawing tools. Collagen III staining was calculated as the ratio of total intensity of strong positive strong (ISP)/the total analyzed area (ISP/Area) for each animal. For K63-ubiquitinated proteins and p16INK4A staining, results are expressed as the ratio: number of strong positive (NSP)/the total analyzed area (NSP/Area).

### 4.8. RNA Extraction from DBA/2J Urinary Samples and Real-Time PCR Analysis

RNA was isolated from cell-free urine using the miRNeasy Serum/Plasma Kit (Qiagen, Hilden, Germany) according to the manufacturer’s instructions by the use of a QIAcube robotic workstation for automated RNA extraction. The exogenous Cel-miR-39 spike-in (Qiagen) was added in equal concentrations to all samples prior to RNA isolation to monitor the efficiency of RNA extraction. Total RNA was eluted in 15 μL of RNase-free water and stored at −80 °C until further use.

Before performing real-time PCR, 2 μL RNA from each sample were retro-transcribed using the miScript II RT Kit and HiSpec Buffer (Qiagen). cDNA was then diluted 10 folds and assayed in 10 μL PCR reactions; miR-27b-3p was assayed in duplicate by qPCR using the miScript SYBR Green PCR Kit and the following primer pair: hsa-miR-27b-3p miScript Primer Assay (cat. No. MS00031668). Amplification was performed using the LightCycler^®^ 96 Real-Time PCR System (Roche Diagnostics, Indianapolis, IN, USA) in 96-well plates. 

### 4.9. ELISA Measurement of Urinary Albumin and Urine Creatinine in DBA/2J Mice

DBA/2J urinary albumin was measured using the Urinary Albumin Mouse ELISA (Abcam). The 24 h albumin excretion was obtained by measuring albumin concentrations in urine and multiplying its value according to the total urinary volume of 24 h per each animal. Data were then normalized to the amount of total protein per sample. DBA/2J mice urine creatinine was measured using the Urinary Creatinine Assay Kit (Cell Biolabs, San Diego, CA, USA) according to the manufacturer’s instructions. Briefly, 5 microliters of each urine sample were diluted 10-fold using deionized water in a 96-well plate. In the same plate, 10 creatinine standard reactions at known concentrations were prepared starting from a 20 mg/dL creatinine stock. The provided creatinine reaction reagent was then added to samples and standards and incubated for 30 min on an orbital shaker. The resulting creatinine-picrate complex was then measured and the absorbance at 490 nm was recorded. Sample creatinine concentrations were determined by comparison with the known creatinine standards.

### 4.10. Statistics

For histological evaluation, animal data were expressed as median ± interquartile range (IQR) and compared with a Mann–Whitney test. A *p* value < 0.05 was considered statistically significant. All analyses were performed by using GraphPad Prism 5.0 (GraphPad software, Inc., San Diego, CA, USA).

For quantitative PCR, amplification curves were analyzed using the Roche LC software, both for melting curve analysis and for determination of Cq by the second derivative method [34]. All assays were inspected for distinct melting curves, and the Tm was checked to be within known specifications for the assay. miRNA expression level was normalized to the cel-miR-39 (Caenorhabditis elegans miR-39) spike-in control and calculated through the equation: ΔCq = Cq target − CqCel39. A *p*-value < 0.05 was considered statistically significant.

## 5. Conclusions

Pre-clinical studies can be leveraged to ameliorate current therapeutic approaches since they allow the identification of novel therapeutic targets [35]. In this scenario, we showed that the inhibition of K63 ubiquitination by NSC697923 could be considered as a novel option to halt the process leading to kidney fibrosis in experimental diabetes. In addition, the additive effect of the association of conventional ACE inhibitor treatment with NSC697923 with regard to a reduction of fibrosis and the potential role of NSC697923 in the reduction of albuminuria as well as its effect on miRNA-27b-3p modulation can represent a novel approach to tackle the multifactorial pathophysiology of renal complication in diabetes. 

## 6. Patents

The methods and results described in the present manuscript are included in patent no. 102018000004126.

## Data Availability

All data generated during this study is included in this published article (and its supplementary information files).

## Supplementary Materials

The following figures are available online at https://www.mdpi.com/article/10.3390/ijms22105194/s1, Figure S1: Cell viability of RPTEC cells in the indicated conditions using Annexin-V and 7AAD staining; Figure S2: Lysine 63 ubiquitinated proteins, staining in female non-diabetic mice. Figure S3: Collagen III staining in female non-diabetic mice. Figure S4: Sirius Red staining in female non-diabetic mice. CTR: control; RAM: Ramipril; NSC: NSC697923. NSP/Area: Number of strong positive/Area.
