# Supplementary material for: Inhibition of Lysine 63 Ubiquitination Prevents the Progression of Renal Fibrosis in Diabetic DBA/2J Mice
Source: Int J Mol Sci. 2021 May 14;22(10):5194. doi: 10.3390/ijms22105194 (PMC8157080; doi:10.3390/ijms22105194)
Supplement: Supplementary file 1 [file ijms-22-05194-s001.zip › ijms-1231208-supplementary.pdf]

Supplementary Figure 1: Cell viability of RPTEC cells in the indicated conditions using Annexin-V and 7AAD staining. HG: high glucose; NSC: NSC697923.

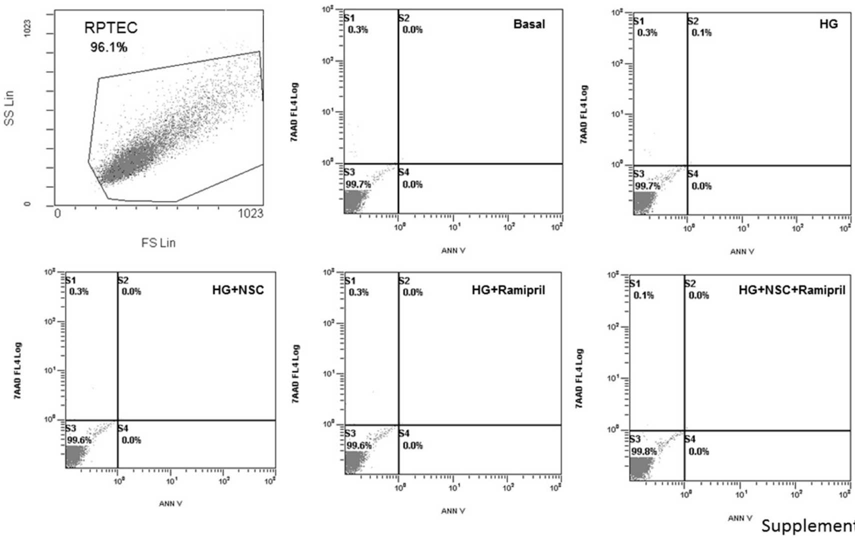

Supplementary Figure 1

Supplementary Figure 2: Lysine 63 ubiquitinated proteins, staining in female non-diabetic mice. CTR: control; RAM: Ramipril; NSC: NSC697923. NSP/Area: Number of strong positive/Area

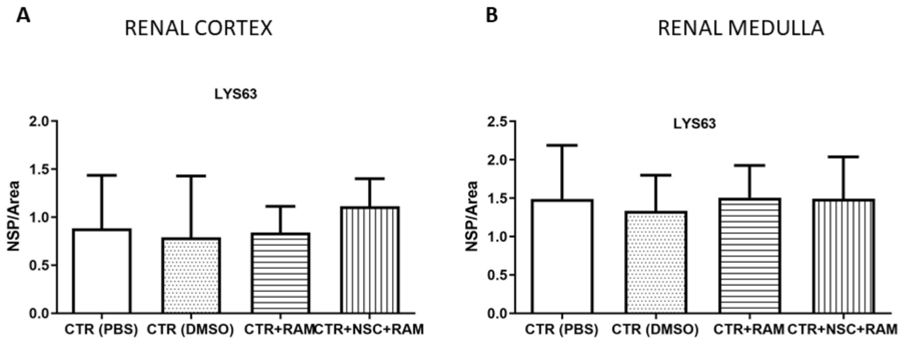

Supplementary Figure 2

Supplementary Figure 3 – Collagen III staining in female non-diabetic mice. CTR: control; RAM: Ramipril; NSC: NSC697923. ISP/Area: Intensity of strong positive/Area

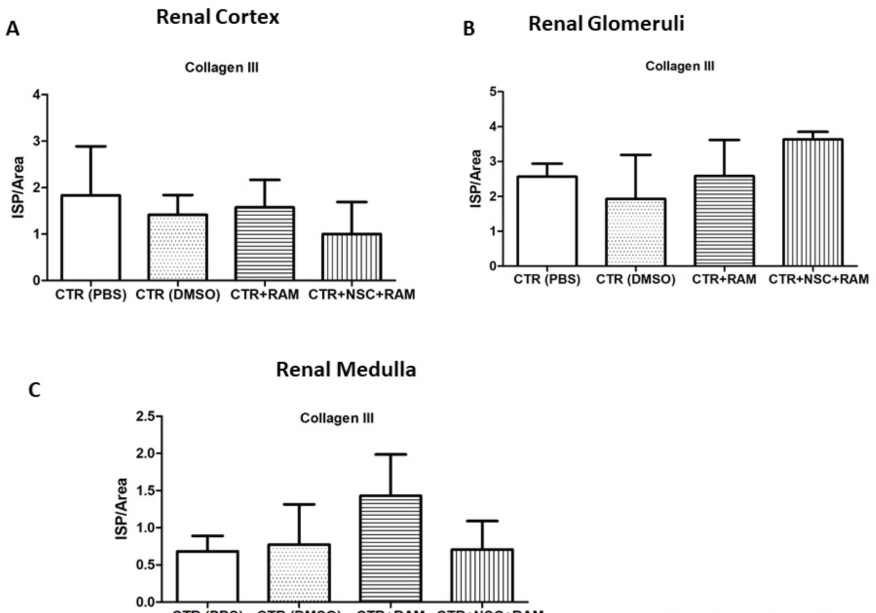

Supplementary Figure 3

Supplementary Figure 4 – Sirius red staining in female non-diabetic mice. CTR: control; RAM: Ramipril; NSC: NSC697923. NSP/Area: Number of strong positive/Area

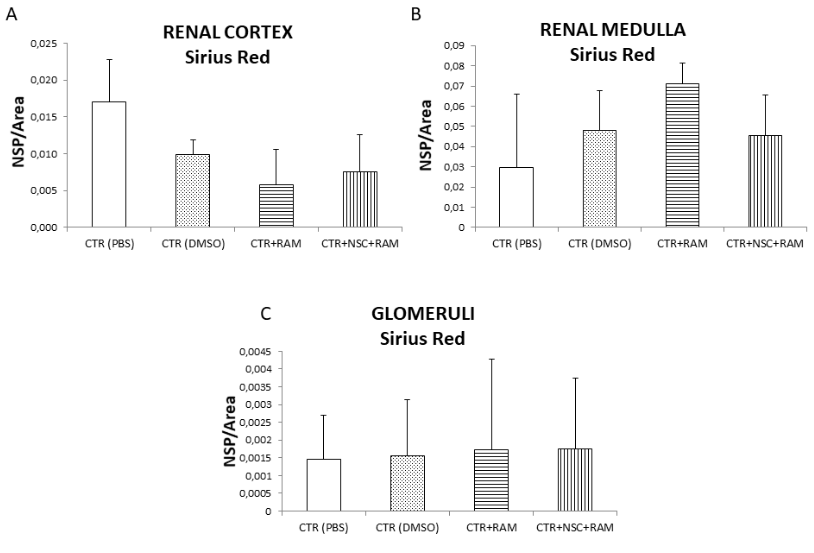

Supplementary Figure 4
